# An Improved Method for Diagnosis of Parkinson’s Disease using Deep Learning Models Enhanced with Metaheuristic Algorithm

**DOI:** 10.21203/rs.3.rs-3387953/v1

**Published:** 2023-10-04

**Authors:** Saurav Mallik, Babita Majhi, Aarti Kashyap, Siddarth Mohanty, Sujata Dash, Aimin Li, Zhongming Zhao

**Affiliations:** harvard public health; Central University; Central University; Central University; Nagaland University; Xi'an University of Science and Technology; The University of Texas Health Science Center at Houston

**Keywords:** Parkinson’s Disease, SPECT DaTscan, T1, T2-weighted, deep learning, VGG16, InceptionV3, Grey wolf optimization

## Abstract

Accurate diagnosis of Parkinson's disease (PD) at an early stage is challenging for clinicians as its progression is very slow. Currently many machine learning and deep learning approaches are used for detection of PD and they are popular too. This study proposes four deep learning models and a hybrid model for the early detection of PD. Further to improve the performance of the models, grey wolf optimization (GWO) is used to automatically fine-tune the hyperparameters of the models. The simulation study is carried out using two standard datasets, T1,T2-weighted and SPECT DaTscan. The metaherustic enhanced deep learning models used are GWO-VGG16, GWO-DenseNet, GWO-DenseNet + LSTM, GWO-InceptionV3 and GWO-VGG16 + InceptionV3. Simulation results demonstrated that all the models perform well and obtained near above 99% of accuracy. The AUC-ROC score of 99.99 is achieved by the GWO-VGG16 + InceptionV3 and GWO-DenseNet models for T1, T2-weighted dataset. Similarly, the GWO-DenseNet, GWO-InceptionV3 and GWO-VGG16 + InceptionV3 models result an AUC-ROC score of 100 for SPECT DaTscan dataset.

## 1. Introduction

Parkinson's disease, also known as neurodegeneration, is an illness characterised by the progressive death of dopamine-producing brain cells. Dopamine is an organic substance produced by neurons that serves as a neurotransmitter in the brain, facilitating communication between neurons. Parkinson's disease results from impaired neuronal communication due to insufficient dopamine production in the brain. The substantia nigra, a small region of the brain, is where the neurons of the human brain are got affected due to Parkinson's disease, a long-term, neurological, and progressive motor illness [1].

A new United Nations research claims that nearly 1 billion people worldwide, or approximately one in six, suffer from neurological conditions like epilepsy, migraine, brain injuries, and neuro-infections like Alzheimer's, PD, stroke, and multiple sclerosis. Each year, 6.8 million of these sufferers were passing away [2]. Although the actual cause of Parkinson's disease is unknown, it is believed that a combination of inherited and environmental factors is responsible for it[3].In the modern world, PD affects 2–3% of people who are at the age of 65 and older [4]. Parkinson's disease progresses differently in every patient, and it is impossible to anticipate how quickly the disease may progress in any specific person. While some people may have only minor symptoms for years, others may do so quite fast as they progress to more severe problems. Parkinson's disease often starts with minor tremors or other motor symptoms on one side of the body and progresses slowly over a number of years. The disease's symptoms could extend throughout the body and get worse, possibly affecting both sides of it. Even though Parkinson's disease is an ongoing and advancing condition, there are medicines that can help to manage symptoms and improve the standard of living. Parkinson's disease does not yet have an appropriate early diagnosis or treatment. Medication, physical therapy, and lifestyle modifications are some of its treatments. Parkinson's disease progression can be slowed down or stopped, even though there is presently no known cure for it.

These days, artificial intelligence (AI) approaches - machine learning (ML) and state of art deep learning (DL) are greatly assisting medical professionals in the early diagnosis of illnesses. Due to this, research has recently been done to automatically identify Parkinson’s disease using MRI images utilising a variety of AI and ML algorithms. Many different diseases and ailments have been diagnosed using deep learning, and the findings frequently outperform traditional benchmarks [5].

Deep learning models which are a state of art performance mostly used in image classification problems With their ability to learn intricate patterns and features from images, they can often surpass traditional machine-learning approaches in accuracy. It automatically extracts relevant features from images, hence cause the elimination of manual feature engineering. This feature extraction capability permits the model to learn complicated representations and capture both low-level and high-level features present in the images. It can handle large-scale datasets efficiently. They can learn from vast amounts of labelled data, which is essential for training accurate image classifiers. Deep learning frameworks and libraries are designed to leverage parallel computing resources like GPUs to accelerate training and inference processes.

Over the past two decades, meta-heuristic optimization techniques have gained a lot of popularity. A few of these include particle swarm optimization (PSO) [6], grey wolf optimization (GWO) [7], ant colony optimization (ACO) [8], artificial bee colony optimization (ABC)[9], etc. Hyperparameter tuning is one of the tedious jobs to manually fine-tune the parameters to obtain the best optimal values. The population-based metaheuristic algorithm known as Grey Wolf Optimisation (GWO) is influenced by the way grey wolves hunt. It searches for the best answers in a problem area by combining exploration (diversification with exploitation (intensification). It is used to automatically fine-tune the parameters which mimic the social behaviour of grey wolves, including their leadership hierarchy and group hunting. The improved capacity of GWO prevents results from being stuck in the local optimal value [10]. It also finds the best solution with a quick convergence rate.

The benefits of deep learning and GWO in image classification include higher accuracy, autonomous feature extraction, scalability, transfer learning skills, robustness to fluctuations, finding the optimal solutions, automatic hyperparameter tunning and continuous progress through continuing research and development. A variety of AI approaches using ML and DL models have been created in the past. In this study, a new framework is employed by combining grey wolf optimization (GWO) with four deep learning models known as VGG16[11], DenseNet [12], InceptionV3[13], DenseNet-LSTM [14] and a hybrid model VGG16 + InceptionV3.

The following is a concise explanation of the paper's main contribution.

- Number of images created empty tuples, for which difficulty is coming while running the deep learning algorithms. These empty tuples are removed to obtain better performance by using the Python function.
- Proposed four deep learning models with hyperparameter optimization by GWO known as GWO-VGG16, GWO-DenseNet, GWO-InceptionV3, and GWO-DenseNet-LSTM.
- Proposed hybrid model using GWO-VGG16 + InceptionV3.
- The proposed models are compared with the existing models using various performance metrics.

Following is the format for the remaining section: The earlier studies are covered in Section 2. Section 3 explains the preprocessing of MRI images and the development of methodologies. Experimental results and discussions, and comparisons between the existing models and proposed models are discussed in Section 4. In Section 5, conclusions and future scope are discussed briefly.

## 2. Related Literature

In the past few years, various studies have been created and published by academics worldwide to help in Parkinson's disease diagnosis. Many of these researchers have used various AI methods to analyse and classify the MRI brain images in order to detect various diseases related to Parkinson’s disease. Deep learning techniques are the most often used method for classifying MRI images due to their capacity to deliver superior results than those obtained by more conventional machine learning techniques. This particular section explains the research using ML and DL methods to diagnose patients with Parkinson’s disease.

### 2.1 Related review literature using T1, T2-weighted dataset

Camacho et al. (2023) [15] have developed a robust explainable deep-learning classifier trained for the classification of Parkinson’s disease using a T1-weighted MRI dataset. A total of 1,024 PD and 1,017 subjects from matched controls (HC) of the same age and gender are gathered from 2,041 MRI data i.e.,T1-weighted MRI datasets from 13 separate investigations. The datasets underwent a skull-stripping process, isotropic resampling, bias field correction, and nonlinear registration to the MNIPD25 atlas. Convolutional Neural Network is trained to categorise PD and HC participants using the Jacobian maps produced from the fields of deformation and fundamental clinical data. [16] provides improved knowledge of the clinical variables associated with Impulse Control Behaviours (ICB) and structural and functional brain abnormalities in PD patients. They have measured grey and white matter brain volume and graph topological features using multimodal MRI data.[17] introduces a new technique for categorising a person's 3-D magnetic resonance scans as a diagnostic tool for Parkinson's disease by using one of the largest Parkinson’s Progressive Marker Initiative (PPMI) MRI datasets from a patient group with the condition and healthy controls. Due to the fact that gender has a substantial impact on neurobiology and PD cases are developed more likely in males than women, it is advantageous that different research is conducted for men and women. [18] have examined the viability and usefulness of employing multi-modal MRI datasets to distinguish between PD, PSP-RS, and HC subjects automatically. For this investigation, there are 45 PD, 20 PSP-RS, and 38 HC subjects with available T1-weighted MRI datasets, T2-weighted MRI datasets, and diffusion-tensor (DTI) MRI datasets. Brain morphology using T1-weighted, brain iron metabolism using T2-weighted, and microstructural integrity using DTI dataset regional values are determined by an atlas-based approach. These values are used to choose features, and then classification is performed using a variety of well-known machine-learning approaches.[19] have proposed a 3D CNN architecture after data pre-processing to learn the complex patterns in MRI images to identify Parkinson's Disease. 406 individuals from the baseline visit, including 203 in good health and 203 with Parkinson's disease, are selected for the experiment.

A novel method is used by [20] which trains a deep neural network model using data from new patients, specifically with T1 MRI and DaTscan datasets. The information utilised to model the knowledge retrieved from the PPMI database contains a set of vectors that represent the clustering centres of these representations, along with the matching DNN structure. The ability of the unified model created using these many datasets to predict Parkinson's disease in an effective and transparent manner has then been demonstrated. [21] has proposed two new deep-learning techniques for ensemble learning-based Parkinson's disease detection. Instead of using the entire MR image, authors focused on the Grey and White Matter areas which greatly improved detection accuracy and obtained 94.7% accuracy. To discover which brain regions are important in the decision-making process for architecture is performed by occlusion analysis as well. Multiple parcellated brain areas are used by [22] to train a CNN. The idea is to create a complicated model by combining the models from various locations using a greedy algorithm. Three retrospective investigations included 305 PD patients (59.9–9.7 years of age) and 227 HC patients (61.0-7.4 years of age). Based on the Automatic Anatomic Labelling template, fractional anisotropy and mean diffusivity are determined and then divided into 90 different brain regions of interest (ROIs).

The authors in [23] have suggested CNN with eight layers deep for 3D T1-weighted MRI images to differentiate between PD and HC individuals. The proposed model additionally made use of the information provided by the individuals' ages and genders. In addition, batch and group normalization are applied to the designed model, increasing the accuracy up to 100%. [24] has described an autonomous diagnosis approach that distinguishes between PD and HC with high accuracy. Benchmark T2-weighted MRI scans for both PD and HC are made available to the public by the PPMI. Image registration technique is used to choose and align the middle 500 slices of a T2-weighted MRI scan.

### 2.2 Related review literature using SPECT DaTscan dataset

Thakur et al. (2022) [12] has constructed a CNN model that can accurately pinpoint the ROIs after feature extraction.1,390 groups of DaTscan images with PD and normal classes are analysed in the paper. The final classification layer includes a soft-attention block which makes use of the DenseNet-121 design. After classifying the images, Soft Attention Maps and feature map representation a reutilized to visually analyse the region of interest (ROI). [25] have worked sought to establish an ensemble deep learning technique with three stages for PD patient prognosis. Retrospective information on 198 Parkinson's disease (PD) patients is obtained from the PPMI database and then randomly 118 patients are assigned to training, and 40–40 patients are assigned to both validation and test set. The features are extracted from DaTscan dataset and clinical assessments of motor symptoms in the steps 1 and 2. And in step 3, an ensemble of DNN is trained to predict 4 years of patient outcome. [26] have created a CNN model that can distinguish between PD patients and HC patients based on SPECT images. In this study, 2723 images of SPECT dataset are used out of which 1364 samples from the PD group and 1359 samples from the HC group. The image normalization method is used to improve the regions of interest (ROIs) required for the network to learn attributes that set them apart from other regions of interest (ROIs). In order to assess the effectiveness of the network model, 10-fold cross validation is used. [27] provide six well-known interpretation techniques and four deep-convolutional neural network designs. Also, the authors suggest a mechanism for evaluating interpretation performance as well as a way to use interpreted input to aid in model selection. [11] suggest a computer learning model that accurately identifies whether every given DaTscan has PD or not while offering a logical justification for the prediction. Visual indicators are created utilising Local Interpretable Model-Agnostic Explainer (LIME) approaches. Further, transfer learning is used to train DaTscans on a CNN (VGG16) from the PPMI database, and the resulting models have 95.2% of accuracy. Finally, the paper concludes that the suggested approach may successfully assist medical professionals in the PD detection because of its measured interpretability and accuracy. To analyse pictures from dopamine transporter single-photon emission computed tomography (DAT-SPECT) has been suggested utilising an ANNin [28]. With the use of an active contour model, striatal regions are segmented and utilised as the data performing transfer learning on the artificial neural network which is pre-trained to distinguish Parkinson’s disease. To serve as a benchmark, support vector machine is trained to use semi-quantitative measurement metrics including the specific binding ratio (SBR) and asymmetry index.

The active contour model is utilized to segment the striatal regions in the images. These segmented regions are then employed as the dataset for an already-trained ANN to do transfer learning. The goal is to separate PD from Parkinsonism associated with other disease. [29]have used artificial neural networks (ANN) and image processing techniques to identify Parkinson's disease in its early stages. The images used are 200 SPECT scans from the PPMI dataset, out of which 130 are of normal participants and 70 are of Parkinson's disease (PD) patients. Using the sequential grass fire algorithm, the caudate and putamen areas of the images are determined. To distinguish healthy and Parkinson's disease-infected people, these above features are loaded into an ANN. A novel approach is introduced by [30] for the medical treatment of neurodegenerative disorders, like Parkinson's, that utilises trained DNNs to extract and utilise latent information. The paper uses transfer learning along with k-means clustering, K-NN classification, and DNN trained representations to enhance disease prediction using MRI data. In recent past, [31] have presented a model for the early identification of PD which combines image processing with ANN in order to improve the imaging diagnosis of PD. The caudate and putamen serve as the study's region of interest (ROI), and the model identified them by analysing 200 SPECT images from the PPMI database, out of which 100 are of healthy people and 100 are of PD people. The ANN is then fed with the ROI area data, with a thought it will recognise patterns similar to how a human observer would do. [32] have suggested a novel method that uses 3-dimensional convolutional neural networks (CNNs) to differentiate between PD and healthy control. In order to reduce overfitting and boost the neural network's generalisation abilities, the training set as well as the data from this set's sagittal plane using a straightforward data augmentation technique is given as input to the model.

One of the difficult challenges all are facing is determining Parkinson's disease in the early stages. To conduct research on the early detection of PD using MRI images, various authors developed numerous computer-based machine learning and deep learning methods as described above.

In this study, authors have proposed four deep learning models whose hyperparameters are optimized using GWO, namelyGWO-VGG16, GWO-DenseNet, GWO-DenseNet + LSTM, GWO-IncepionV3, and a hybrid model (GWO-VGG16 + InceptionV3) which is the novelty of this paper. No authors earlier used these models with T1, T2-weighted and SPECT DaTscan for PD detection. Here, a number of images are creating empty tuples, for which difficulty is coming while running the deep learning algorithms. These empty tuples are properly handled and removed to obtain better performance. This problem has also never been addressed by any authors previously in the literature.

## 3. Materials and Methods

**Figure 1:**
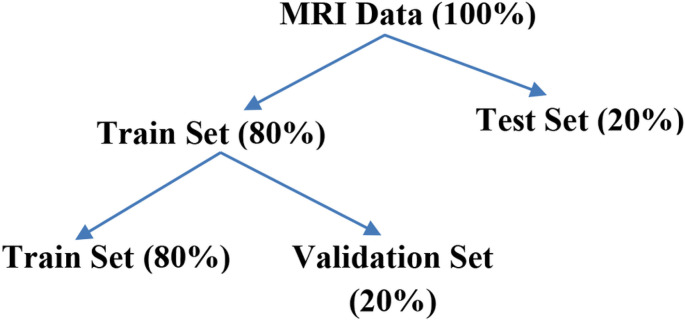
Splitting of MRI datasets.

This section illustrates the proposed methodology, preprocessing of MRI images, and model development. After that, the data is divided into two sets using 80:20 ratio for the train and test sets. Again, the train set is divided into train and validation sets. The 80% of input images are fed to the proposed model for training and then the models are validated using 20% from the train set samples. Finally, models are tested using the remaining 20% of the data. The distribution is depicted in Fig. 1 below.

### 3.1 Proposed Methodology

In the proposed methodology following are the steps :

Step 1: Firstly, T1, T2-weighted and SPECT DaTscan MRI datasets are collected from the PPMI website.

Step 2: MRI images are then pre-processed using preprocessing techniques such as the conversion of DICOM file to .jpg format, cropped images using Micro DICOM Viewer desktop application, removing empty tuples and finally, skull stripping is done using python packages “simple ITK”. Normalization is also done using batch normalization for scaling.

Step 3 : Datasets are divided into train and test sets using the holdout method (80:20 ratio) Again train set is divided into (80:20 ratio) two sets i.e. train and validation set.

Step 4: Four deep learning models are proposed whose hyperparameters are optimized by GWO, known as GWO-VGG16, GWO-DenseNet, GWO-DenseNet-LSTM, GWO-InceptionV3, with one hybrid model GWO-VGG16 + InceptionV3.

Step 5: Finally, results are evaluated using various performance measures such as accuracy (acc), sensitivity (sen), specificity (spe), precision (pre), f1_score(f1-scr) and AUC score.

**Figure 2:**
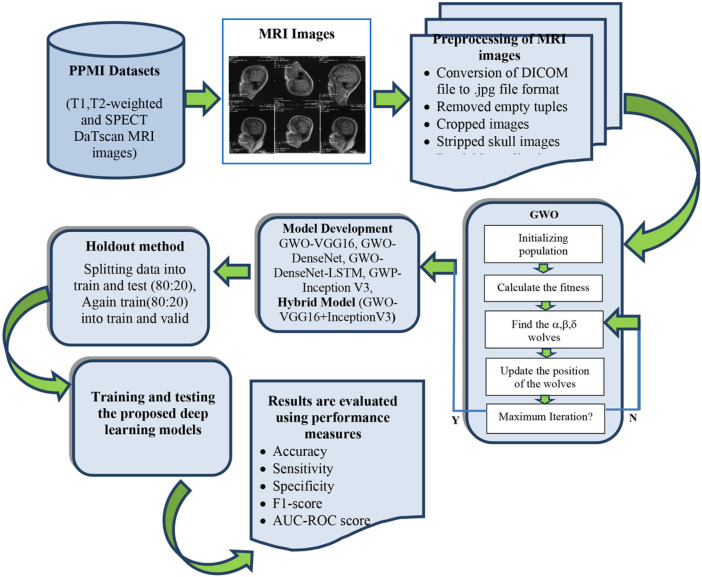
Proposed methodology for early detection of PD using GWO and deep learning models.

The proposed methodology is also graphically presented in Fig. 2.

### 3.2 MRI data collection

The MRI data are extracted from PPMI website [33]. The PPMI dataset is a large-scale longitudinal investigation of Parkinson's Disease (PD) conducted by the Michael J. Fox Foundation for the research of Parkinson's. The objective of the study is to find biomarkers that can aid in predicting the onset and progression of PD and to create new treatments for the condition. The PPMI dataset contains a variety of information, including clinical evaluations, genetic information, biospecimen samples (blood and CSF), and brain imaging data (MRI and DaTscan). Researchers from all across the world can analyse and do research on the dataset.

One of the distinguishing characteristics of the PPMI dataset is its longitudinal nature, which monitors patients over a number of years. This feature enables researchers to examine changes in disease development and find potential biomarkers for the illness. The dataset also includes a large control group of healthy individuals, which provides a baseline for comparison. T1, T2-weighted MRI [34] and SPECT DaTscan [33] datasets used in this study are collected from the PPMI website.

### 3.2 MRI Data Samples

In this study, two datasets are used i.e. T1, T2-weighted and SPECT DaTscan. A total 30 number of subjects are included in T1, T2-weighted MRI dataset, from which 15 subjects (Male-7, Female-8) have Parkinson’s disease (PD) and 15 subjects (Male-7, Femal-8) are healthy control (HC), which contains a total number of 9070 MRI images of different sizes. Out of 9070 MRI images, 3620 are PD subjects, and 5450 are HC subjects. A total 36 number of patients are included in the SPECT DaTscan dataset, from which 18 subjects (Male-9, Female-9) are suffering from Parkinson’s disease (PD) and 18subjects (Male-9, Female-9) are healthy control (HC) which contains a total of 20096 MRI images. Out of 20096 MRI images, 14344 are PD subjects and 5752 are HC subjects. The sample size is distributed as shown in Table 1.

#### Inclusion Criteria

Those patients are included in the study whose age is between 55 and 75 years. Only PD and HC subjects are included.

#### Exclusion Criteria

Patients whose age is less than 55 and greater than 75 are excluded from this study. Other category subjects are excluded such as SWEDD, PRODROMAL, etc.

### 3.3 Image Pre-processing

MRI images are available in DICOM (Digital Imaging and Communications in Medicine) [35] file format which is used to store and send medical pictures like X-rays, CT scans, and MRIs. A lot of image-related metadata, including patient data, information on the image's acquisition, and other medical data, is included in DICOM files. However, the DICOM five format is difficult to deal with when employing these pictures for machine learning tasks.

Many machine learning libraries and frameworks don't natively support DICOM files, which is one of the reasons DICOM images are generally transformed to other image formats, like png or jpg, before being used for image classification. Although Python has libraries for reading and manipulating DICOM files, it can often be simpler to convert the images to a more widely used format, such as png or jpg, and then use conventional image processing packages to work with the images.

Another reason for converting DICOM images to jpg is that DICOM images have different pixel representations and bit depths, depending on the specific equipment and software used to generate them. Jpg images, on the other hand, have a standardized pixel representation and bit depth, making them more consistent and easier to work with.

Finally, unlike some other picture formats, png, jpg images don't lose any information when they are compressed, which might be crucial in the area of medical imaging, where even minor data loss can have serious repercussions.

**Figure 3:**
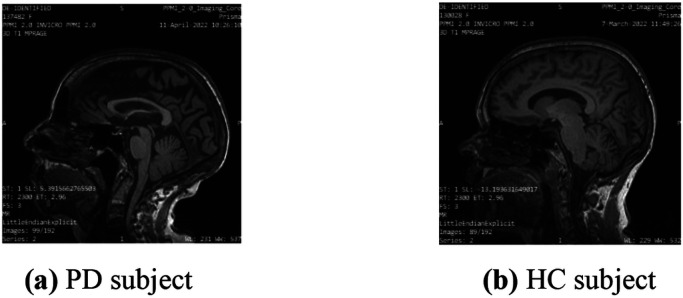
Original MRI brain images of T1,T2-weighted dataset (a) PD subject (b) HC subject.

In this study, all theDICOM (.dic) file format images are first converted into the .jpg format using MicroDICOM Viewer desktop application. The original image size is 256 x 256 x 3. The images which generate empty tuples are removed from the selected images. Empty tuples are those which created the null arrays for which the machine learning models create a huge number of misclassifications. These images are removed based on the threshold value of 30 pixels. Then images are cropped and stripped using python library functions. After it, images are normalized using batch normalization. After preprocessing, final size of the MRI images is 224 x 224 x 3which given as input to the models. The original MRI images are shown in Figs. 3 (a) and 3 (b).

**Figure 4:**
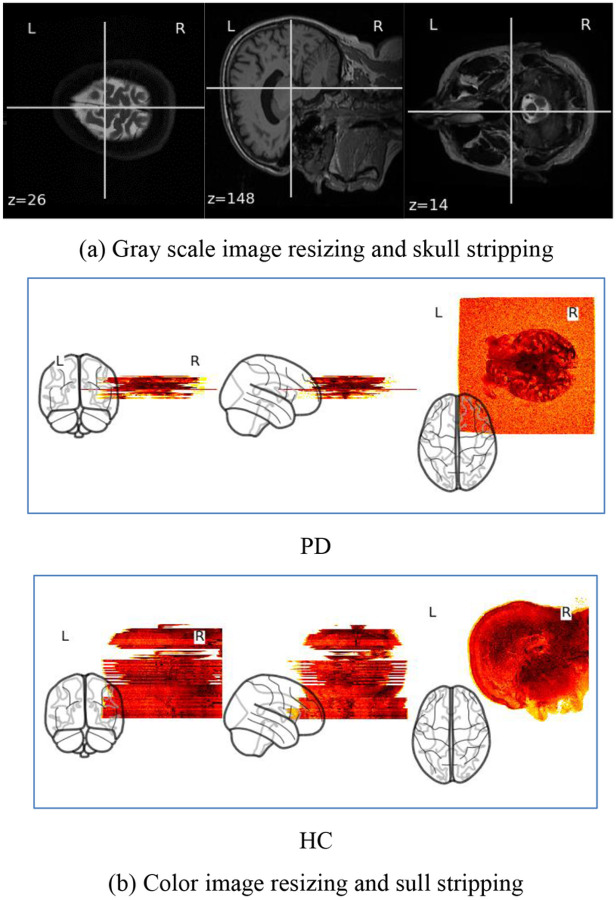
Image resizing and skull stripping of T1,T2-weighted datasetof both thePD and HC subjects in (a) gray scale and in (b) color.

After pre-processing the images are shown in Figs. 4(a) and (b).

### 3.4 Model development

Four deep learning models with the combination of grey wolf optimization technique GWO-VGG16, GWO-DenseNet, GWO-DenseNet-LSTM, GWO-InceptionV3 and a hybrid model GWO-VGG16 + InceptionV3 have been proposed in this study for detection of PD accurately. All the proposed models are explained briefly below:

#### VGG16

VGG16 (Visual Geometry Group 16) [11]is a deep CNN architecture that has suggested by the University of Oxford's Visual Geometry Group in 2014.It is created for image classification problems and has accomplished state-of-the-art performance on various benchmarks, including the ImageNet Large Scale Visual Recognition Challenge (ILSVRC) dataset. Thirteen Conv (convolutional) layers, 3 fully connected dense layers, and other layers made up the 16-layer,VGG16. The input layer accepts an image as input of size 224 × 224×3. Each of the 13 convolutional layers is having3x3 filters with a stride of (1). After each max pooling layer, the number of filters doublesi.e.6 x 6 with a stride of 2, starting with the first convolutional layer that includes 64 filters. The max pooling layers help to decrease the number of model parameters and avoid overfitting by reducing the spatial dimensions of the output by a factor of 2. Padding is a technique that is used by all convolutional layers to guarantee that the output's spatial dimensions match those with the inputs. Rectified linear unit (ReLU) is one of the activation functions that introduces nonlinearity into the model comes after each convolutional layer. It has 2 fully connected layers, each with 256, 128neurons respectively. There are 128 neurons in the output layer, corresponding to the two classes in the T1,T2-weighted and SPECT DaTscan datasets. In order to output a probability distribution over the classes, it uses a "sigmoid" activation function. The VGG16 algorithm is renowned for its ease of use and capacity to extract intricate information from images. However, it can be expensive to train and utilise computationally because it is a very deep network with huge parameters.

#### DenseNet

DenseNet[12], short for Dense CNN, is a deep learning architecture that Huang et al. have first presented in 2016. It is designed to address the vanishing gradient problem and encourage deep neural networks that reuse features. It creates connections that are dense between all layers. Each layer in this architecture receives feature maps from all levels below it as input. Gradient flow throughout the network is made possible by this connection structure, which provides direct access to features at various depths.

DenseNet is made up of dense blocks, each of which has several levels. Each layer in a dense block is connected to all layers before it. The overall network design is created by gradually connecting dense units. Convolutional and pooling layers are employed as transition layers to shorten the distance between packed blocks. They contribute to preserve connections while lowering computational complexity and feature map sizes. The key advantages of DenseNet are feature reuse, parameter efficiency, and mitigating the vanishing gradient problem.

DenseNet is widely used and has produced state of the art outcomes for a number of computer vision applications, such as semantic segmentation, image classification and object recognition. It is now a well-liked option among deep learning researchers and practitioners.

#### DenseNet-LSTM

DenseNet with LSTM [14] refers to a network that combines Long Short-Term Memory (LSTM) networks with the DenseNet network. The strengths of LSTM's modelling of sequential data and ability to detect temporal relationships are combined with DenseNet's feature extraction skills in this hybrid architecture.

The DenseNet component serves as the feature extraction backbone. The dense connections and hierarchical structure aid in the efficient acquisition of both local and global image features. At various degrees of abstraction, the DenseNet layers process the input image or sequence to extract significant information.

Afterwards, an LSTM network receives the output from the DenseNet layers. The LSTM is a form of recurrent neural network (RNN) that excels at modelling sequential data because it preserves long-term dependencies and detects temporal patterns. Memory cells are present in the network, allowing it to recall or forget information over time selectively. DenseNet and LSTM are used in various applications such as video action recognition, natural language processing, sentiment analysis, etc in order to identify actions or activities, DenseNet captures features from individual frames, while LSTM processes the sequence of features.

#### InceptionV3

InceptionV3[13] is a variant of the Inception architecture that is introduced by Christian Szegedyet al. in 2015. InceptionV3 is a deep neural network that is created for image classification and object detection tasks. It consists of an input layer, stem network, inception modules, auxiliary classifiers, average pooling, fully connected dense layers and a final (output) layer.

The images are given as input to the input layer, typically of size 224x 224 x 3. The stem network extracts features from the input images using three convolutional layers. With a 3x3 kernel, the first, second and third layer consist of 32, 32 and 64 filters, respectively. The max pooling layer, which follows the stem network, has a 3x3 filter with a stride of 2.

There are several inception modules in InceptionV3 that are responsible to do feature extraction at various scales. Each inception module is made up of a number of convolutional layers with pooling layers and of various filter of sizes (1x1, 3x3, and 5x5) concatenated along the channel dimension. Compared to conventional convolutional layers, Inception modules are computationally inexpensive. Two auxiliary classifiers are included in InceptionV3 after the 5th and 9th inception modules. The auxiliary classifiers are made up of a dropout layer, a softmax activation function, a ReLU activation function, a fully connected layer with 1024 neurons, and a global average pooling layer. The auxiliary classifiers' role includes supplying the network with more training data and minimizing the vanishing gradient issue.

After the last inception module, InceptionV3utilizes a global average pooling layer to shrink the output's spatial dimensions to 1x1 feature map. A fully connected layer with 128 neurons is fed with the output of the global average pooling layer, which corresponds to the two classes in the T1, T2-weighted and SPECT DaTscan datasets. The fully connected layer outputs a probability distribution over the classes using a sigmoid activation function.

#### Proposed Hybrid Model (VGG16 + InceptionV3)

**Figure 5:**
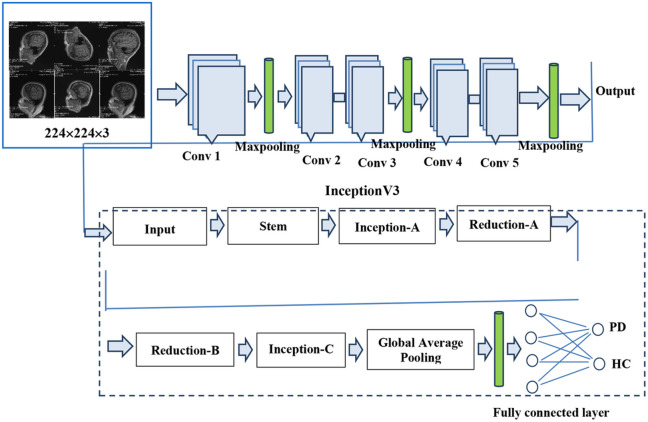
Architecture of the proposed Hybrid Model (VGG16+InceptionV3) for PD detection.

The combination of VGG16 and InceptionV3 is known as a hybrid model. Both models are described above individually. The VGG16 model's output is used as input to the InceptionV3 model, and finally, it foretells whether a given patient will get Parkinson's disease or not. Figure 5 illustrates the hybrid model's entire architecture.

#### Grey Wolf Optimization (GWO)

**Figure 6:**
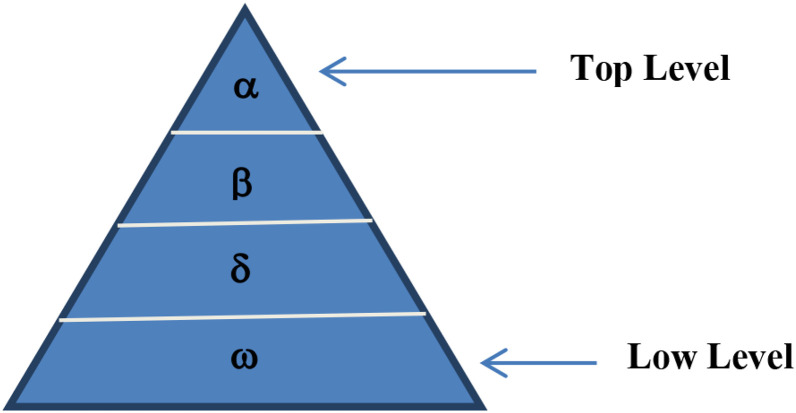
The hierarchy of grey wolf optimization top to bottom.

Seyedali Mirjalili has introduced GWO in 2014 by imitating the social conduct, hierarchy of leadership, and hunting on the communal land of grey wolves [7]. Canidae is the family that includes the grey wolf (Canis lupus). As the top predators in the food chain, grey wolves are known as apex predators. The majority of grey wolves prefer to live in packs. The typical size of the group is between 5 and 12 people. Alpha, Beta, Delta and Omega are four different species denoted by (α), (β), (δ) and (ω) and as shown in Fig. 6.

The step-by-step procedure of grey wolf hunting is as follows:

1. Tracking, chasing, and approaching the prey.
2. As soon as the target starts moving, it is pursued, hounded, and surrounded.
3. attacking the prey or assaulting it.

In this section, social hierarchy, encircling, and attacking is mathematically represented as follows

#### Social hierarchy

Alpha is the best solution (α) to mathematically express the social hierarchy, followed by (β) and (δ) as the next two best options. The remaining candidate solution is the (ω). α, β, and δ serve as the hunting (or optimisation) cues in the GWO algorithm. The remaining ω wolves come after these α, β, and δwolves.

#### Encircling/Surrounding Prey

Grey wolves circle their prey during hunting. The encircling behavior is mathematically represented as

(1) $$V=|\vec{S}.\vec{T}{\;}_{{}_{x}}(t)-\vec{T}\;(t)|$$

(2) $$\vec{T}\;(t+1)=\vec{T}{\;}_{{}_{x}}(t)-\vec{U}.\vec{V}$$

where current iteration is denoted by t, coefficient vectors are denoted by S and U, the position of the prey is denoted by T_x_, and grey wolf's position is denoted by T. The vector $\vec{S}$ and $\vec{U}$ are represented as

(3) $$\vec{U}=2\vec{p}.\vec{q_{1}}-\vec{p}$$

(4) $$\vec{S}=2.\vec{q_{2}}$$

where $q_{1}$, $q_{2}$are arbitrary vectors with a range of [0, 1] and the components of $\vec{p}$ decrease linearly from the value 2 to 0 throughout the course of iterations.

#### Hunting the prey:

**G**rey wolves have the ability to track down and encircle their prey. Typically, the alpha leads the hunt. Hunting may occasionally be done by the beta and delta. It is assumed that the most promising candidate solution, alpha, delta, improves knowledge of the potential prey’s location in order to replicate the hunting behaviour of the wolves mathematically. The three best candidate solutions are mathematically represented to update their position as follows –

Alpha Wolf, Beta Wolf, Delta Wolf

(5) $$\vec{V}{\;}_{\alpha }=|\vec{S}{\;}_{1}.\vec{T}{\;}_{\alpha }-\vec{T}|$$

(6) $$\vec{V}{\;}_{\beta }=|\vec{S}{\;}_{2}.\vec{T}{\;}_{\beta }-\vec{T}|$$

(7) $$\vec{V}{\;}_{\delta }=|\vec{S}{\;}_{3}.\vec{T}{\;}_{\delta }-\vec{T}|$$

(8) $$\vec{T}{\;}_{1}=\vec{T}{\;}_{\alpha }-\vec{U}{\;}_{1}.\left(\vec{V}{\;}_{\alpha }\right)$$

(9) $$\vec{T}{\;}_{2}=\vec{T}{\;}_{\beta }-\vec{U}{\;}_{2}.\left(\vec{V}{\;}_{\beta }\right)$$

(10) $$\vec{T}{\;}_{3}=\vec{T}{\;}_{\delta }-\vec{U}{\;}_{3}.\left(\vec{V}{\;}_{\delta }\right)$$

(11) $$\vec{T}\;(t+1)=\frac{\vec{T}{\;}_{1}+\vec{T}{\;}_{2}+\vec{T}{\;}_{3}}{3}$$

The wolves in motion attack the prey when it stops. $\vec{U}$ is a randomly chosen number between – 2r and 2r, while r_2_ is a number between – 1 and 1. The search agent's next position is a position that falls somewhere between the object's most recent location and its preyer position. Thus, the attacking state is appropriate when $|\vec{U}|<1$ [36]. The behaviour of wolves used to depict the process of finding the best solution. Following is the pseudocode of gray wolf optimization:

#### Pseudocode for the grey wolf optimization -

Initialize population of grey wolves as Zi, where, i = 1,2,…..n

Initialize p, U and S

Fitness calculation for every search agent

Z _α_ = Best search agent

Z _β_ = 2nd best search agent

Z _δ_ = 3rd best search agent

While (t < Max_iterations)

For every search agent

Update the current position using Eq. (11)

$$\vec{T}\;(t+1)=3\vec{T}{\;}_{1}+\vec{T}{\;}_{2}+\vec{T}{\;}_{3}$$

End of the for loop

Update the value of p, U and S.

Fitness calculation of all search agents then

Update Z _α_, Z_β_and Z_δ_

t = t + 1

end of while loop

return Z _α_

#### Algorithm for hyperparameters optimization of deep learning models using GWO:

Step-by-step procedure:

Step-1 : Set the ranges of hyperparameter values. The ranges are given in Table 3.

Step-2 : Set the population size of the grey wolves.

Step-3 : Create an objective function that measures how well the deep learning models performed after being trained with the provided hyperparameters. This function gauges the model's effectiveness on a validation set.

Step-4 : Using the wolves' fitness levels, the dominance and hierarchy are determined.

Step-5 : Update the alpha, beta and delta and omega wolf's position using the Eq. (11)

Step-6 : Make that the wolf’s new positions remain inside each hyperparameter's stated ranges. A location is modified if it exceeds the limits.

Step-7 : Verify that the termination condition-such as completing the required number of iterations or obtaining the target fitness value-is met. The optimisation procedure ends if the condition is satisfied; otherwise, return to step 5.

Step-8 : Take the optimal collection of hyperparameters for the deep learning models, which corresponds to the solution that is best and represents the wolf with the highest fitness value.

## 4. Experimental Results and Discussion

Prior to discuss the outcomes, the fundamental performance evaluation criteria that are frequently used to evaluate different machine learning models while they are still in the training phase as well as in the testing phase are discussed in this section.

### 4.1 Performance evaluation with confusion matrix

The confusion matrix [37] which is a two-dimensional table is used to determine performance metrics. It displays the actual and predicted class values which are represented by its elements as true positive (T + ve), true negative (T−ve), false positive (F + ve), and false negative (F−ve). Checking the degree of misunderstanding between the various classes can be done by calculating these four elements. Based on the confusion metrics, the five score metrics used in this study are as follows –

(12) $$\text{Accuracy (Acc)}=\frac{TP+TN}{TP+TN+FP+FN}\;\ldots$$

(13) $$\text{Sensitivity (Se)}=\frac{TP}{TP+FN}\;\ldots$$

(14) $$\text{Specificity (Spe)}=\frac{TN}{TN+FP}\;\ldots$$

(15) $$\text{Precision (P)}=\frac{TP}{TP+FP}\;\ldots$$

(16) $$\text{F1-score}=2\times \left(\frac{Precision\times Recall}{Precision+Recall}\right)[37]\ldots$$

Along with the true + ve rate and false + ve rate, the ROC curve is shown on a graph which is known as receiver operator characteristic. Area under curve (AUC) score, or area under the curve, is also obtained.

### 4.2 Experimental Results analysis of all the proposed models

Experimental results are described by the following subsections where it displayed the training results by plotting the accuracy and loss curves for each model used. For each model, the confusion matrix is also created and displayed.

#### 4.2.1 Training Results of all the proposed Deep Leaning Models

The experiments are carried out in Python by using various packages such as keras, opencv, tensorflow 2.1, scikitlearn [38] using the system configuration of intel Core i5 processor, 8th Generation, with 16 GB RAM, and NVIDIA GEFORCE graphics combined with 8 GB memory. The standard T1, T2-weighted and SPECT DaTscan datasets are used for the study. The datasets are split into two sets i.e., train and test using an 80:20 ratio. Again, the training set is then split into train and validation sets. To train all the proposed deep learning models with GWO using the algorithm given in section 3.4, various hyperparameters used are shown in the Table 2 and the optimized hyperparameters by GWO are shown in Table 3.

All the proposed deep learning models GWO-VGG16, GWO-DenseNet, GWO-DenseNet-LSTM, GWO-InceptionV3 and hybrid model GWO-VGG16 + InceptionV3 are pre-trained using the above hyperparameters. All the models comprise of an input layer, two hidden layers and an output layer. Every model has their own layers, such as convolutional, max-pooling, stem, global average pooling etc. Each layer consists of 256, 128 number of neurons respectively. Every hidden layer ends with a dropout layer with 20 percent of neurons dropping out to overcome the overfitting problem. ReLu activation function is employed to all the hidden layers. To train all the models ‘adam’ optimizer and loss function ‘binary cross entropy’ is used. The GWO algorithm is used for hyperparameters optimization with all the proposed models to obtained the better performance. The ranges of parameters are given manually and optimized hyperparameters are shown in the Table 3.

##### Training/Validation accuracy/loss for T1, T2-weighted MRI Dataset

**Figure 7:**
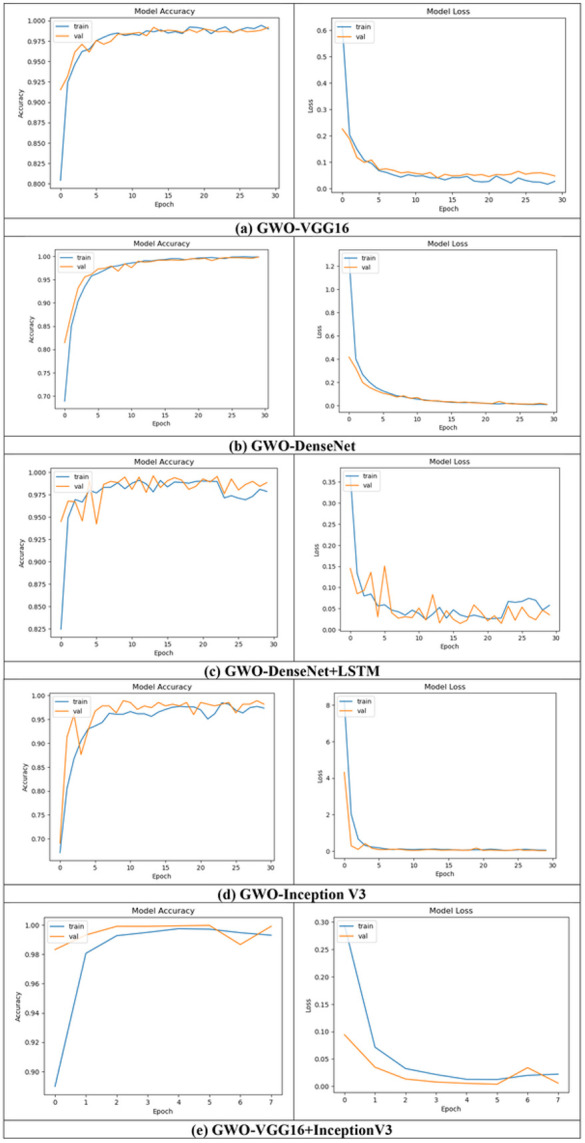
Plots for accuracy/loss during training and validation of all the proposed models (a) GWO-VGG16, (b) GWO-DenseNet, (c) GWO-DenseNet-LSTM, (d) GWO-InceptionV3 and (e) Hybrid model (GWO-VGG16+InceptionV3) using T1,T2-weighted MRI dataset.

**Figure 8:**
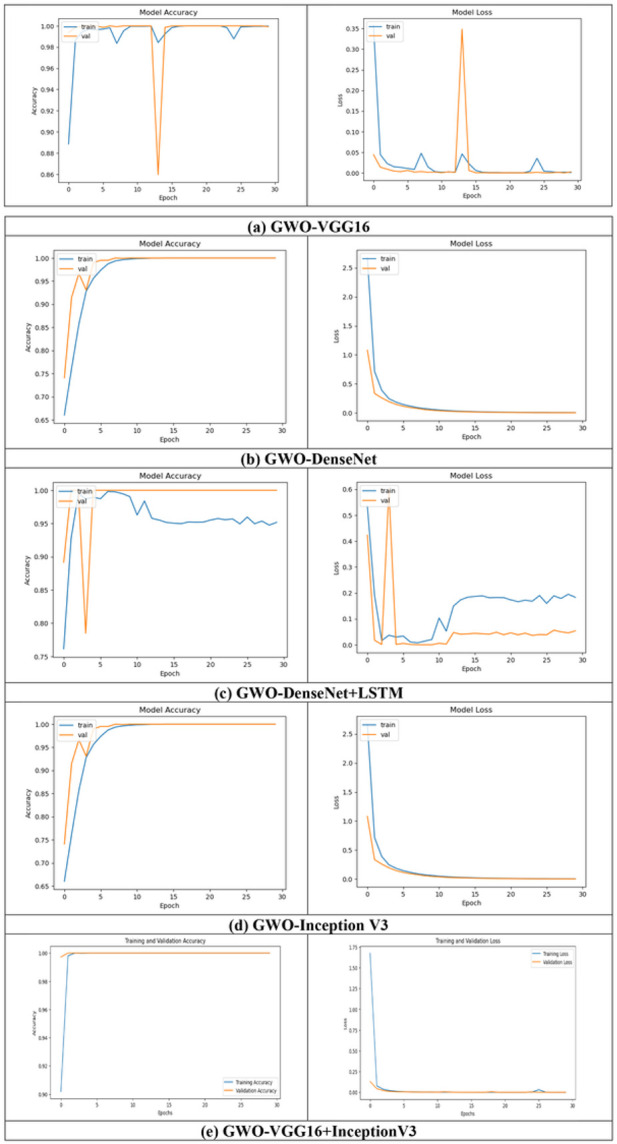
Plots for accuracy/loss during training and validation of all the proposed models (a) GWO-VGG16, (b) GWO-DenseNet, (c) GWO-DenseNet-LSTM, (d) GWO-InceptionV3 and (e) Hybrid model (GWO-VGG16+InceptionV3) using SPECT DaTscan dataset.

The training accuracy and training loss are plotted for all the proposed models for both the T1,T2-weighted MRI and SPECT DaTscan datasets are exhibited in Figs. 7(a)-(e) and Figs. 8(a)-(e), respectively.

It is clearly observed from the above figures that the optimized model’s accuracy in some models initially fluctuates but the curve becomes smoother as the model is taught/trained more. Additionally, it can be seen from the loss plot that GWO provides a superior loss rate in some models than the others.

Training/Validation accuracy/loss for SPECT DaTscan Dataset is shown Fig. 8.

The x axis shows curves, and the y axis represents improvement. How well a model is trained using train set is shown by the training curve. In actuality, only 30 epochs of convergence are sufficient for all models. The validation curves revealed whether a particular model is underfitting, overfitting, or just correct for a particular range of hyperparameter values. Additionally, there is very little overfitting in all of the models. In light of this, the accuracy of convergence on training set is quite close to the accuracy of convergence on validation set. Here, it is observed that at the validation phase all models performed well and obtained more than 99% accuracy.

#### 4.2.2. Testing Results of all the proposed Deep Leaning Models

**Figure 9:**
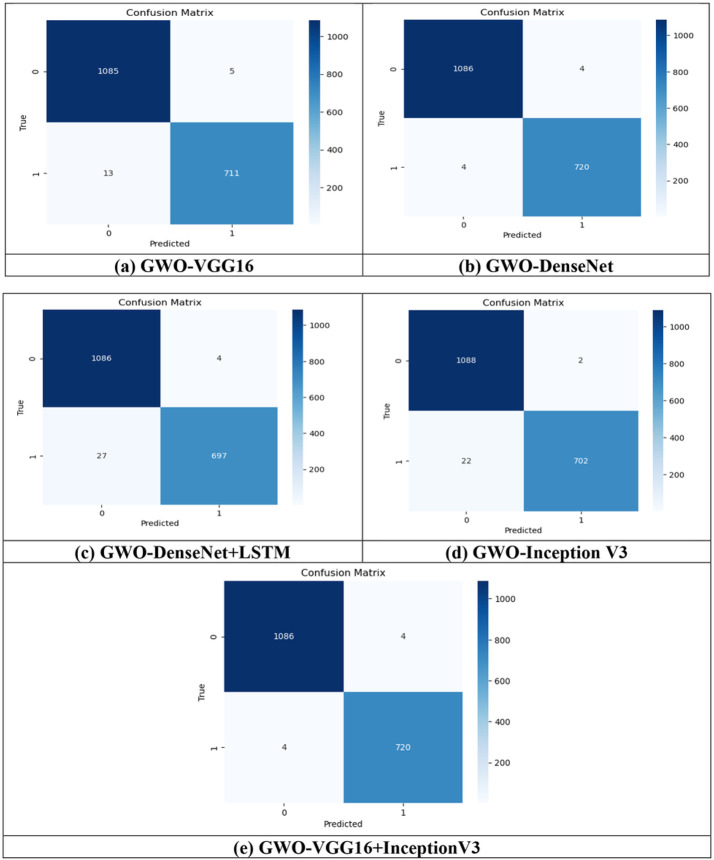
Confusion matrix for all the proposed models (a) GWO-VGG16, (b) GWO-DenseNet, (c) GWO-DenseNet-LSTM, (d) GWO-InceptionV3 and (e) Hybrid model (GWO-VGG16+InceptionV3) using T1,T2-weighted MRI dataset.

**Figure 10:**
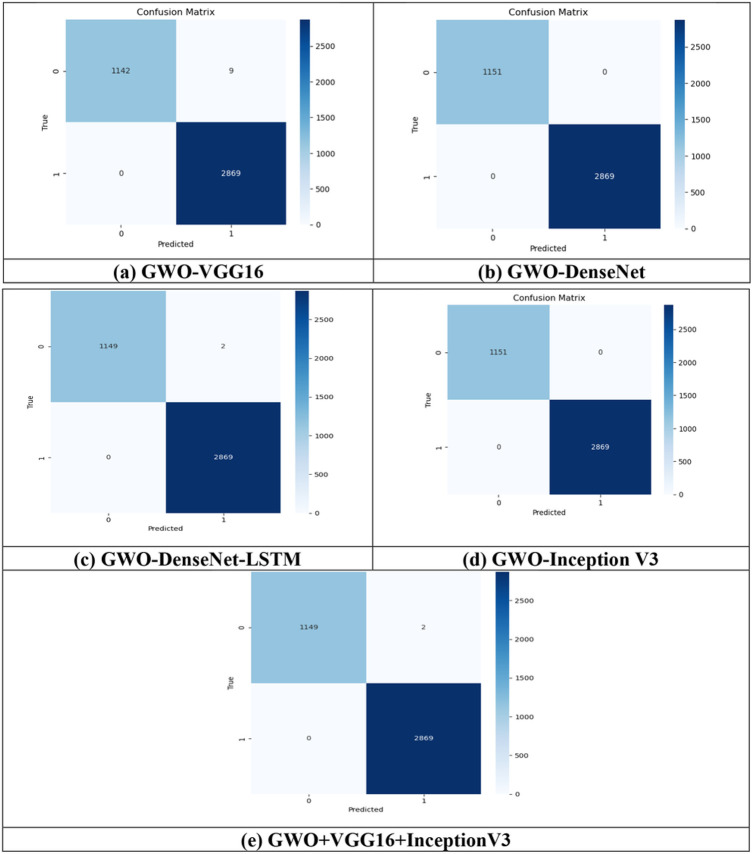
Confusion matrix for all the proposed models of all the proposed models (a) GWO-VGG16, (b) GWO-DenseNet, (c) GWO-DenseNet-LSTM, (d) GWO-InceptionV3 and (e) Hybrid model (GWO-VGG16+InceptionV3) using SPECT DaTscan dataset.

A fully separate data subset that is previously prepared, is used to test and evaluate the effectiveness of the proposed models. The Figs. 9 (a)-(e) and Figs. 10 (a)-(e) show the confusion matrix for all the proposed models for both the datasets i.e., T1,T2-weighted MRI and SPECT DaTscan, respectively.

Confusion Matrix using T1, T2-weighted MRI dataset is shown is Fig. 9.

Confusion Matrix using SPECT DaTscan dataset is shown in Fig. 10.

#### 4.2.3. Experimental Results and Discussions

Before obtaining the results of the proposed models, various preprocessing techniques and hyperparameter optimization technique is applied to obtain better accuracy and other performance measure results. Four deep learning models VGG16, DenseNet, DenseNet + LSTM, InceptionV3 and a hybrid model VGG16 + InceptionV3are trained using the two standard datasets T1, T2-weighted and SPECT DaTscan with 9070 and 20096 images for 30 epochs. The results are briefly explained below for both the datasets.

##### Results using T1,T2-weighted MRI dataset

The results evaluation and comparison of all the proposed models are presented in the Table 4 and the ROC curve is plotted for each model.

**Figure 11:**
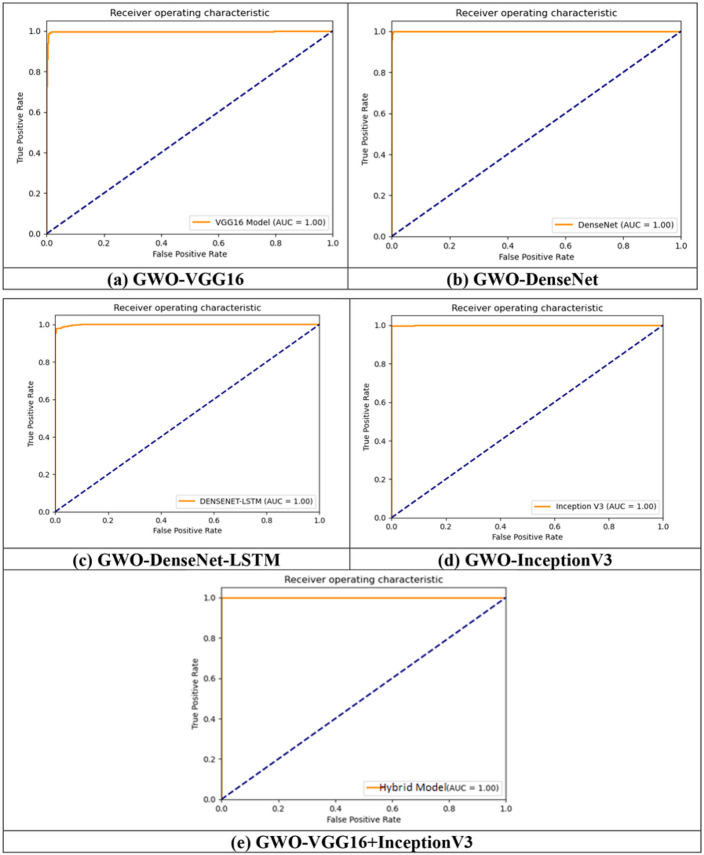
ROC plot for all the proposed models (a) GWO-VGG16, (b) GWO-DenseNet, (c) GWO-DenseNet-LSTM, (d) GWO-inceptionV3 and (e) Hybrid model (GWO-VGG16+InceptionV3) using T1,T2-weighted dataset.

The results of Table 4 demonstrate that all the proposed models achieved more than 99% of testing accuracy except GWO-DenseNet + LSTM which resulted 98.29% accuracy and the hybrid model (GWO-VGG16 + InceptionV3) obtained highest accuracy 99.94% with the training loss of 0.0272 which is minimum among all models. To examine these results, the ROC curve showing the diagnostic test's sensitivity (sen) vs. specificity(spe)are used. This kind of curves aid in comparing various models depending on the value of the AUC variable. For each model, ROC curve is plotted and given in the Figs. 11(a)-(e) which presents the curve between the true + ve rate (TPR) and false + verate (FPR).

Based on the representation of ROC curve, all the proposed models obtained AUC of approximately 100%. This proves that the proposed models are best in detection of Parkinson’s disease.

#### 4.4.2 Results using SPECT DaTscan MRI dataset

The result evaluations and comparison of all the proposed models are given in Table 5 and the ROC curve is plotted for each model for DaTscan datasets.

**Figure 12:**
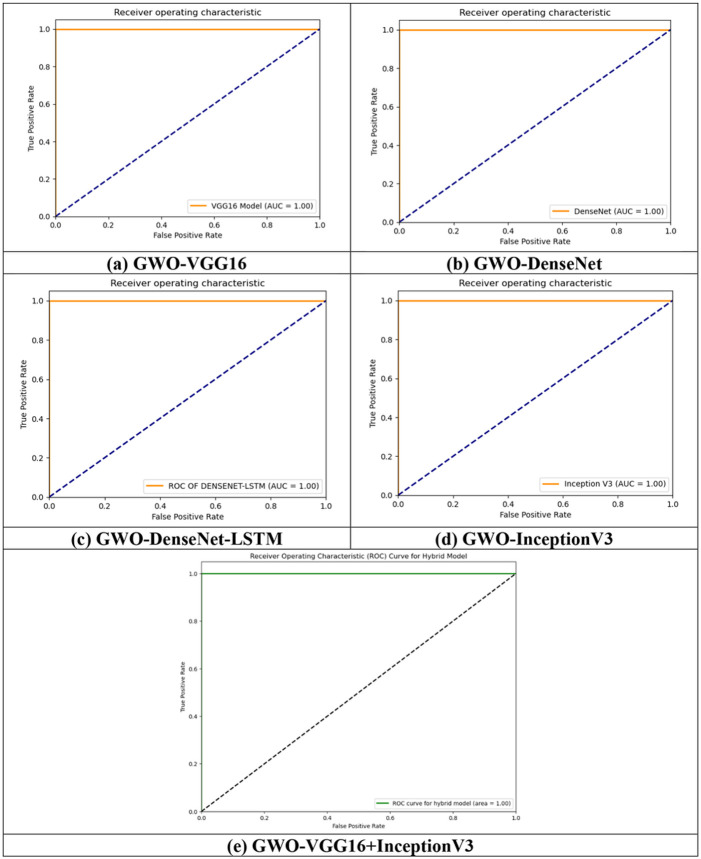
ROC plot for all the proposed models (a) GWO-VGG16, (b) GWO-DenseNet, (c) GWO-DenseNet-LSTM, (d) GWO-InceptionV3 and (e) Hybrid model (GWO-VGG16+InceptionV3) using SPECT DaTscan dataset.

The aforementioned table demonstrates that all the proposed models achieved approximately 100% testing accuracy. The GWO-DenseNet, GWO-InceptionV3 and hybrid model GWO-VGG16 + InceptionV3 obtained exactlyl 100% of testing accuracy with the training loss value 0.0038, 0.0942 and 0.0153 respectively. For each model, ROC curve is plotted in the Figs. 12(a)-(e) which represents the curve between the true + ve rate (TPR) and false + verate (FPR). All the models obtained approximately 100% of AUC.

### 4.3 Comparison with the existing models

The proposed models' outcomes are presented in Tables 6 and 7 along with comparisons to other previously reported models. The comparison exhibit that for both datasets, the proposed deep learning models beat all other existing models in terms of performance metrics like accuracy, sensitivity, specificity, precision, f1-score and AUC score.

**Figure 13:**
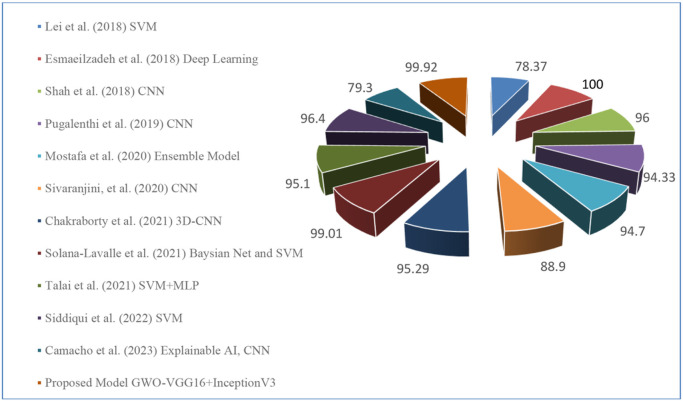
Pie chart representation of comparison between proposed deep learning model with other existing models using T1,T2-weighted dataset.

The above table shows that, in terms of accuracy, from all the proposed deep learning models, hybrid model GWO-VGG16 + InceptionV3 outperform the other eleven existing models and obtained 99.92% accuracy which is nearly similar to the model proposed by [23] with 100% accuracy. The comparisons are graphically represented by pie chart as shown in Fig. 13.

**Figure 14:**
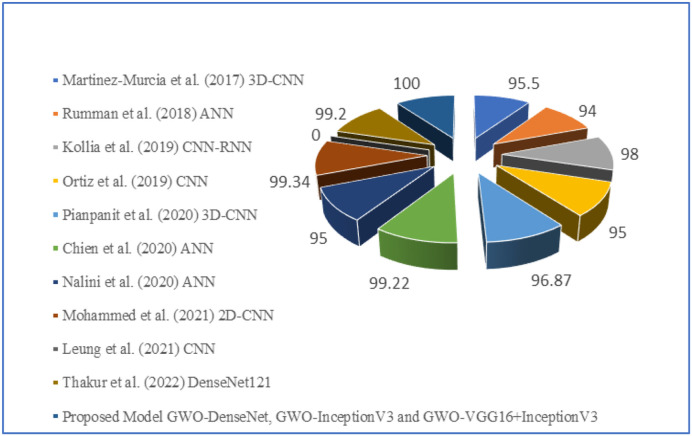
Pie chart representation of comparison between proposed deep learning model with other existing models using SPECT DaTscan dataset.

Table 7 shows that in terms of accuracy, from all the proposed models, GWO-DenseNet, GWO-InceptionV3 and hybrid model GWO-VGG16 + InceptionV3 outperform the other existing models with 100% of accuracy. The comparisons are graphically represented by pie chart and given in Fig. 14.

The study has certain research limitations. Firstly, limited sample size is taken for the experiment; bigger sample size is still required to confirm the validity of these experimental models. Besides that, to implement with the larger sample size, it needs high memory spaces more than 16GB RAM, high-end GPU system in this case. Because of the high dimension data, it takes more time to execute and increases the time and space complexity. Only two datasets T1, T2-weighted MRI and SPECT DaTscan are used in this study. Only binary class classification problem is used for early prediction of the Parkinson’s disease. Multiclass classification can also be done.

## 5. Conclusion and Future Work

The detection of Parkinson's disease is becoming more and more crucial today. Because PD is a tremor illness, it is increasingly difficult to make an accurate diagnosis of the condition, especially in the early stages. This study proposes a classification approach for Parkinson's disease (PD) detection that enables doctors to make an accurate and timely diagnosis. The paper proposes four deep learning models, VGG16, DenseNet, DenseNet + LSTM, InceptionV3 and a hybrid model VGG16 +InceptionV3. In order to optimise the proposed deep learning models technique their performance in handling the PD problem, the GWO algorithm is used to automatically tune the hyperparameters. Two datasets T1, T2-weighted and SPECT DaTscan are used for the experiment. Various preprocessing techniques are applied to the images to enhance the models' functionality. After pre-processing, the GWO optimization algorithm is fitted into all the models and efficiently encoded. The model’s efficiency is measured by comparing it with the existing models. The outcome shows that compared to other models; the suggested/proposed model has demonstrated superior accuracy. using both datasets. Finally, GWO-VGG16 + InceptionV3 obtained 99.92% accuracy in T1,T2 dataset and GWO-DenseNet, GWO-InceptionV3 and GWO-VGG16 + InceptionV3 obtained 100% accuracy in SPECT DaTscan dataset. The advantages of developing these models, is that it saves doctors time by assisting them as well as cut down the lengthy clinical tests.

The work can be expanded in the future by adding new hyperparameter tuning techniques. Feature extraction will be done using Region of Interest (ROI) of two regions caudate and putamen in SPECT DaTscan dataset. Segmentation will be applied to the MRI images to detect the Parkinson’s disease. Also, work can be done on multiclass classification problem and more no of patients with larger MRI images.

## Figures and Tables

**Table 1:** MRI data samples distribution

Datasets Used	Total no. of subjects		Total no. of image samples used		Training samples (80%)		Testing (20%)	
					Training samples (80%)	Validation samples (20%)		
T1, T2-weighted MRI images	**30**		**9070**		**7256** (PD-2896, HC-4351)		**1814**	
	PD	15	PD	3620	5805	1451	PD	724
	HC	15	HC	5450			HC	1090
SPECT DaTscan	**36**		**20096**		**16077** (PD-11475, HC-4601)		**4019**	
	PD	18	PD	14344	12862	3215	PD	1151
	HC	18	HC	5752			HC	2869

**Table 2:** Hyperparameters used in all the proposed models

Models	Hyperparameters	Values
GWO-VGG16	No. of hidden layers	2
GWO-DenseNet	No. of dense units in 1st, 2nd	128, 64
GWO-DenseNet-LSTM	Dropout	0.20
GWO-InceptionV3	Activation Function	ReLu
Hybrid (GWO-VGG16 + InceptionV3)	Output Layer	Sigmoid
	Optimizer	Adam
	Loss Function	binary_crossentropy

**Table 3:** Optimized hyperparameters using GWO

Models	Manually tunning (range)	Learning Rate	Batch- size	Momentum	Dense- units	epochs
GWO-VGG16	Lr=[0.001,0.01]	0.001	128	0.9	256, 128	30
GWO-DenseNet	Batch_size=[32, 128]	0.001	128	0.9	256, 128	30
GWO-DenseNet-LSTM	Momentum=[0.9, 0.99]	0.01	128	0.95	256, 128	30
GWO-InceptionV3	Dense_units=[128,512]	0.001	128	0.9	256, 128	30
GWO-VGG16 + InceptionV3	Epochs=[10,50]	0.01	128	0.99	256, 128	30

**Table 4:** Results of the proposed models using T1, T2-weighted dataset

Performance Measures	Proposed Models				
	GWO- VGG16 (%)	GWO- DenseNet (%)	GWO- DenseNet + LSTM (%)	GWO- InceptionV3 (%)	Hybrid Model
					GWO-VGG16 + InceptionV3 (%)
Accuracy	99.00	99.55	98.29	99.77	**99.94**
Sensitivity	98.20	99.44	96.27	100	100
Specificity	99.54	99.63	99.63	99.77	99.21
Precision	99.30	99.44	99.43	99.96	99.84
F1-Score	98.74	99.44	97.82	99.92	99.68
AUC-ROC	99.67	99.99	99.88	99.78	99.99
Training Loss	0.0272	0.009	0.575	0.0348	0.0272

**Table 5:** Results of the proposed models using SPECT DaTscan dataset

Performance Measures	Proposed Models				
	GWO- VGG16 (%)	GWO- DenseNet (%)	GWO- DenseNet + LSTM (%)	GWO- InceptionV3 (%)	Hybrid Model
					GWO-VGG16 + InceptionV3 (%)
Accuracy	99.77	100	99.75	100	**100**
Sensitivity	100	100	99.64	100	99.82
Specificity	99.21	100	99.85	100	100
Precision	99.84	100	99.90	100	99.99
F1-Score	99.68	100	99.87	100	100
AUC-ROC	99.99	100	99.73	100	99.92
Training Loss	0.0221	0.0038	0.0452	0.0942	0.0153

**Table 6:** Proposed models comparison with existing models using T1,T2-weighted dataset

Authors	Models used in their study	Accuracy (%)	Sensitivity (%)	Specificity (%)	Precision (%)	F1- score (%)	AUC- ROC score (%)
Leiet al. (2018) [39]	SVM	78.37	84.70	-	66.73	70.21	94.20
Esmaeilzadeh et al. (2018) [23]	Deep Learning	100	-	-	-	-	-
Shah et al. (2018) [24]	CNN	96	-	-	-	-	-
Ramamurthy et al. (2019) [40]	CNN	94.33	97.47	82.54	95.45	-	-
Mostafa et al. (2020) [21]	Ensemble Model	94.70	-	-	-	-	-
Sivaranjini, et al. (2020) [22]	CNN	88.90	-	-	-	-	-
Chakraborty et al. (2021) [19]	3D-CNN	95.29	94.3	94.30	92.7	93.6	98
Solana-Lavalle et al. (2021) [17]	Logistic, RF, NB, Baysian Net, KNN, MLP and SVM	Men (99.01)	99.35	100	100	-	-
		Women (96.97)	100	96.15	97.22	-	-
Talai et al. (2021) [18]	SVM + MLP	95.1	-	100	-	100	-
Siddiqui et al. (2022) [41]	SVM	96.4					
Camacho et al. (2023) [15]	Explainable AI, CNN	79.3	77.7	81.3	80.2	-	87
**Proposed Model**	**GWO-VGG16 + InceptionV3**	**99.92**	**99.81**	**100**	**99.9**	**100**	**100**

**Table 7:** Proposed models’ comparison with the existing models using SPECT DaTscan dataset

Authors	Models used in their study	Accuracy (%)	Sensitivity (%)	Specificity (%)	Precision (%)	F1- score (%)	AUC- ROC (%)
Martinez-Murcia et al. (2017) [32]	3D-CNN	95.5	96.2	-	-	-	-
Rumman et al. (2018) [31]	ANN	94	100	88	-	-	-
Kollia et al. (2019) [30]	CNN-RNN	98	-	-	-	-	-
Ortiz et al. (2019) [42]	CNN	95	95.5	94.8	-	-	97
Pianpanit et al. (2020) [27]	3D-CNN	96.87	97.10	97.89	-	-	-
Chien et al. (2020) [28]	ANN	99.22	81.8	88.6	-	-	-
Nalini et al. (2020) [29]	ANN	95	-	-	-	-	-
Mohammed et al. (2021) [26]	2D-CNN	99.34	99.04	99.63	-	-	-
Leung et al. (2021) [25]	CNN	-	-	-	-	-	84
Thakur et al. (2022) [12]	DenseNet121	99.2	99.2	99.4	-	99.1	99
**Proposed Model**	**GWO-DenseNet,**	**100**	**100**	**100**	**100**	**100**	**100**
	**GWO-InceptionV3**	**100**	**100**	**100**	**100**	**100**	**100**
	**GWO-VGG16 + InceptionV3**	**100**	**99.82**	**100**	**99.99**	**100**	**99.92**

## Data Availability

The data that has been used in this paper is freely available and can be provided on request.
